# New Books

**Published:** 2008-10

**Crop Biosecurity: Assuring our Global Food Supply**

M.L. Gullino, J. Fletcher, A. Gamliel, J.P. Stack, eds.

New York:Springer, 2008. 148 pp. ISBN: 978-1-4020-8477-5, $74.95

**Economics and Management of Climate Change: Risks, Mitigation and Adaptation**

Bernd Hansjürgens, Ralf Antes, eds.

New York:Springer, 2008. 310 pp. ISBN: 978-0-387-77352-0, $119

**Encyclopedia of Ecology. 5 vols.**

S.E. Jorgensen, Brian Faith, eds.

St. Louis, MO:Elsevier, 2008. 3,120 pp. ISBN: 978-0-444-52033-3, $1,995

**Environmental Change and Human Security: Recognizing and Acting on Hazard Impacts**

P.H. Liotta, David A. Mouat, William G. Kepner, Judith M. Lancaster, eds.

New York:Springer, 2008. 480 pp. ISBN: 978-1-4020-8550-5, $99.95

**Environmental Futures: The Practice of Environmental Scenario Analysis**

J. Alcamo, ed.

St. Louis, MO:Elsevier, 2008. 212 pp. ISBN: 978-0-444-53293-0, $170

**Epidemiologic Studies of Veterans Exposed to Depleted Uranium: Feasbility and Design Issues**

Committee on Gulf War and Health: Updated Literature Review of Depleted Uranium, Institute of Medicine

Washington, DC:National Academies Press, 2008. 60 pp. ISBN: 978-0-309-1206-7, $21

**Estimating Mortality Risk Reduction and Economic Benefits from Controlling Ozone Air Pollution**

Committee on Estimating Mortality Risk Reduction Benefits from Decreasing Tropospheric Ozone Exposure, National Research Council

Washington, DC:National Academies Press, 2008. 229 pp. ISBN: 978-0-309-11994-8, $49

**Falling for Science: Objects in Mind**

Sherry Turkle, ed.

Cambridge, MA:MIT Press, 2008. 232 pp. ISBN: 978-0-262-20172-8, $24.95

**Francis Crick: A Biography**

Robert Olby

Woodbury, NY:Cold Spring Harbor Laboratory Press, 2009. 450 pp. ISBN: 978-0-8796-9798-3, $39

**Inequalities in Young People’s Health: Health Behaviour in School-Aged Children**

Currie, C., Gabhainn, S.N., Godeau, E., Roberts, C., Smith, R., et al.

Geneva:WHO Press, 2008. 220 pp. ISBN: 978-9-2890-7195-6, $40

**Insatiable Curiosity: Innovation in a Fragile Future**

Helga Nowotny

Cambridge, MA:MIT Press, 2008. 216 pp. ISBN: 978-0-262-14103-1, $30

**Land Change Science in the Tropics: Changing Agricultural Landscapes**

Andrew Millington, Wendy Jepson, eds..

New York:Springer, 2008. 274 pp. ISBN: 978-0-387-78863-0, $109

**Neither Gods Nor Beasts: How Science Is Changing Who We Think We Are**

Elof A. Carlson

Woodbury, NY:Cold Spring Harbor Laboratory Press, 2008. 180 pp. ISBN: 978-0-8796-9786-0, $29.00

**Public Participation in Environmental Assessment and Decision Making**

Thomas Dietz, Paul C. Stern, eds.

Washington, DC:National Academies Press, 2008. 360 pp. ISBN: 978-0-309-12543-7, $43

**Statistics for Terrified Biologists**

Helmut van Emden

Hoboken, NJ:John Wiley and Sons, Inc., 2008. 360 pp. ISBN: 978-1-4051-4956-3, $39.95

**Theory and Mathematical Methods in Bioinformatics**

Shiyi Shen, Jack A. Tuszynski

New York:Springer, 2008. 445 pp. ISBN: 978-3-540-74890-8, $179.95

**The Energy Trail—Where It Is Leading**

George H. Croy

Hackensack, NJ:World Scientific Publishing Co., Inc., 2008. 150 pp. ISBN: 978-981-281-857-7, $24

**The Entropy Crisis**

Guy Deutscher

Hackensack, NJ:World Scientific Publishing Co., Inc., 2008. 130 pp. ISBN: 978-981-277-968-7, $48

**The New Public Health, 2nd ed.**

Theodore H. Tulchinsky, Elena A. Varavikova

St. Louis, MO:Elsevier, 2008. 696 pp. ISBN: 978-0-12-370890-8, $79.95

**The Socio-Economic Causes and Consequences of Desertification in Central Asia**

Roy Behnke, ed.

New York:Springer, 2008. 254 pp. ISBN: 978-1-4020-8543-7, $65.49

